# Association Between the Triglyceride–Glucose Index and Incident Chronic Severe Pain in Middle‐Aged and Older Chinese Adults: A Nationwide Cohort Study

**DOI:** 10.1155/prm/2464060

**Published:** 2026-01-30

**Authors:** Dizhou Zhao, Yuanyuan Cui, Hong Lu, Haoyi Yang, Weijie Guo, Zhiming Shan, Chaoran Wu

**Affiliations:** ^1^ Department of Anesthesiology, Shenzhen People’s Hospital, The First Affiliated Hospital, Southern University of Science and Technology, Shenzhen, China, sustc.edu.cn; ^2^ Funan Community Health Service Center, The Eighth Affiliated Hospital, Sun Yat-sen University, Shenzhen, China, sysu.edu.cn

**Keywords:** CHARLS, chronic severe pain, insulin resistance, TyG index

## Abstract

**Background:**

Insulin resistance (IR) has been linked to chronic severe pain (CSP) in previous studies. The triglyceride–glucose (TyG) index serves as an indicator of IR. However, whether TyG control level affects the further CSP incidence has not been well established. In this study, we aimed to identify the association between TyG control level and the risk of CSP.

**Methods:**

Participants with a continuous statement of severe pain in two consecutive waves of investigation from the China Health and Retirement Longitudinal Study were defined as CSP. The TyG control level was divided into four classes. The association between the baseline TyG and TyG control level CSP incidence was analyzed through logistic regression and restricted cubic spline analysis.

**Results:**

A total of 113 (3.18%) of 3546 participants were diagnosed as CSP within 9 years. The TyG index has a significant correlation with high CSP incidence (OR = 1.51 (95% CI, 1.16–1.96), *p* = 0.020). Further research analyzed the trajectory of TyG changes. After adjusting for various confounding factors, comparing to Class 1 with the best control of TyG, the OR for Class 4 with the worst control was 2.30 (95% CI, 1.01–5.25) with *p* = 0.048. In restricted cubic spline regression, the relationship between the TyG index and cumulative TyG and CSP is linear.

**Conclusions:**

The TyG index should be regarded as a simple indicator of CSP incidence. Continuous monitoring of the TyG index is more suitable for predicting CSP incident risk compared to a one‐time TyG measurement, and long‐term, well‐controlled management of the TyG index may be an efficient approach to reduce the risk of CSP incidence.

## 1. Introduction

Chronic severe pain (CSP) is a long‐lasting and refractory pain syndrome. The global statistical prevalence of CSP is about 3% and shows an increasing trend [1–3]. Despite substantial advancements in pharmaceutical treatments and interventional therapies in recent years, patients with CSP always suffer from severe physical pain due to the lack of effective treatment and heavy economic burdens [4]. Thus, early identification of individuals at high risk of CSP will help prevent the progression of the condition.

Several factors have been identified as risk factors associated with CSP incidence, such as smoking, alcoholism, sleep disorders, mental health status, obesity, and metabolic syndrome [5]. Insulin resistance (IR), a common glucose metabolism abnormality syndrome, was previously proven to be associated with the occurrence of CSP, since a poorly controlled IR pathological state will always lead to chronic systemic inflammation and metabolic disturbance, general etiologies of CSP [6, 7]. Thus, early identification of individuals with IR will be beneficial to the prediction and prevention of CSP incidence.

In the clinic, commonly used indicators for IR diagnosis and evaluation include the homeostasis model assessment of insulin resistance (HOMA‐IR), the Matsuda index, and the triglyceride–glucose (TyG) index [8]. Since it was first reported in 2008 [9], the TyG index, which is calculated as Ln [fasting triglycerides (mg/dL) × fasting glucose (mg/dL)/2], has emerged as a convenient and reliable marker for assessing IR. Compared to other indicators, the TyG index takes advantage of (1) the TyG index incorporates both triglyceride and fasting glucose data in its calculation, and the abnormal increase in these two factors is the common reflection of IR [10] and (2) the data for triglycerides and fasting glucose are included in an individual’s general health checkups, making the TyG index easily accessible and suitable for widespread use as a screening and monitoring metric [9]. Moreover, accumulating studies have identified the TyG index as an associated biomarker of lipid/glucose metabolic‐related diseases, such as coronary artery disease, carotid plaque, coronary artery calcification, and acute coronary syndrome [11, 12]. However, considering that there is a proven association between IR and CSP occurrence [13], whether the level of TyG control influences further CSP incidence is still not clearly understood. Here, in this study, we aim to investigate the association between the TyG index and the risk of CSP incidence in individuals by using data from the China Health and Retirement Longitudinal Study (CHARLS).

## 2. Methods

### 2.1. Study Design and Population

Our study was based on data from CHARLS, a long‐term national survey of retired Chinese seniors [14]. The first CHARLS survey was conducted in 2011 (as Wave 1), with follow‐ups conducted approximately every two years thereafter, totaling five waves to date (Wave 2 in 2013, Wave 3 in 2015, Wave 4 in 2018, and Wave 5 in 2020). The CHARLS datasets can be accessed for download on the official CHARLS website (https://charls.pku.edu.cn/en). Ethical approval for data collection was granted by the Biomedical Ethics Review Board of Peking University (IRB00001052‐11015). All participants have signed a consent form.

**FIGURE 1:**
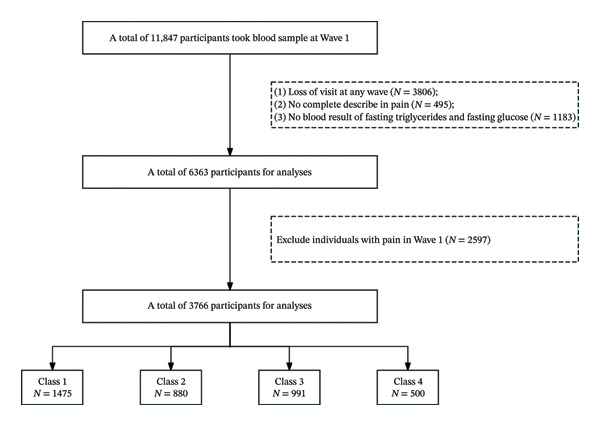
Flowchart of the study population.

In this study, 11,847 participants who underwent a complete blood count test in Wave 1 were included in the analyses. We then excluded 5484 participants for the following reasons: (1) loss of follow‐up in any waves (*N* = 3806); (2) lack of complete description of pain (*N* = 495); and (3) no blood result of fasting triglycerides and fasting glucose in neither Wave 1 nor Wave 3 (*N* = 1183). Then, participants who self‐reported existing pain (declared “Currently Feel Any Body Pains” in the questionnaire) in Waves 1 and 3 were excluded (*N* = 2817). Finally, a total of 3546 participants were included for further analyses (Figure 1.).

### 2.2. Definition of CSP

In general, the International Association for the Study of Pain (IASP) defines chronic pain as persistent or recurrent pain lasting longer than 3 months [2]. Moreover, pain intensity is usually evaluated by the Visual Analog Scales test, where a VAS score ≥ 8 is defined as severe pain.

The definition of CSP in this study was operationalized based on the available data from CHARLS. Pain intensity was assessed using a single‐item question, “Are you troubled with body pain?” We defined “severe pain” as a score of 4 or 5 on the 5‐point scale, referring to a VAS score ≥ 8. While the IASP defines chronic pain as lasting ≥ 3 months, our definition was necessarily shaped by the structure of the CHARLS survey. To identify a subtype of persistent, high‐impact pain, we operationalized CSP as reporting severe pain in two consecutive waves, a definition that leverages the multiyear interval between surveys to capture long‐term severity. This definition intends to capture individuals suffering from severe pain that is sustained over a long period (years), which likely represents a state of significant and disabling chronic pain.

### 2.3. Calculation of TyG Index and Cumulative TyG Index

The TyG index was calculated as Ln [fasting triglycerides (mg/dL) × fasting blood glucose (mg/dL)/2] [8]. Moreover, the CHARLS survey only reported blood measurements data in Wave 1 and Wave 3 investigations. Thus, the cumulative TyG index is calculated as (TyG Wave 1 + TyG Wave 3)/2 × time (2015–2011) [15].

### 2.4. Data Collection and Ascertainment of Covariates

**FIGURE 2:**
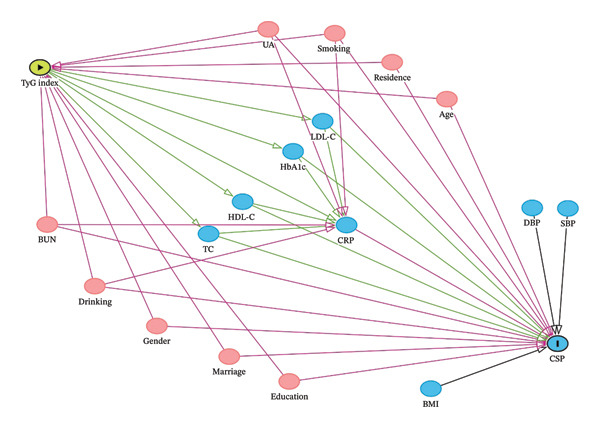
DAG illustrating the assumed causal relationship.

Demographic covariates, blood, and physical measurements data were collected from the baseline questionnaire in Wave 1. Based on medical knowledge and previous studies, we constructed a directed acyclic graph (DAG) to distinguish confounders from mediators (shown in Figure 2). The covariates adjusted for were age, gender, education level, marital status, residence, smoking status, drinking status, blood urea nitrogen (BUN), and uric acid (UA), which were identified as confounders of both the TyG index and CSP. Meanwhile, variables such as lipid profiles, glucose metrics, blood pressure, BMI, and inflammatory markers were identified as mediators. To accurately estimate the total effect of the TyG index on CSP, these mediators were not adjusted for in the primary models.

### 2.5. Statistical Analyses

All the experimental data were analyzed with SPSS (27.0), STATA/SE 16.0, and R Version 4.3.0. *p* < 0.05 was considered to indicate statistical significance. The mean and standard deviation (SD) of continuous data and the percentage of classified data are used to describe basic features. In order to utilize the data as much as possible, we used multiple imputations for missing values, and the number of missing values will be enumerated in the supporting information in Table S1. Continuous variables were compared by ANOVA test or *t*‐test, and categorical variables were compared by chi‐square test or Fisher’s exact probability test. Univariate binary and multivariable logistic regression were used to assess the association between CSP incidence and baseline TyG index in Wave 1. The k‐means clustering method is used for TyG index‐based group classification [16]. CSP incidence was explained using binary logistic regression analysis with odds ratios (ORs) and 95% confidence intervals (CIs). Both univariate binary logistic regression and multivariable logistic regression analysis between CSP incidence and cumulative TyG index, which was adjusted for age, gender, and so on, were used. Restricted cubic splines were used to explain the nonlinear association between CSP incidence and both of the TyG index and the cumulative TyG index. Subgroup binary logistic regression analyses were used in age (cutoff as 65 years ago), gender, education level (primary school or lower, secondary school or higher), residence (agriculture, others), marriage status, SBP (cutoff from normal SBP standards: < 90 mmHg [normal], 90–139 mmHg [elevated], and ≥ 140 mmHg [hypertension]), DBP (cutoff from normal DBP standards: < 60 mmHg [normal], 60–89 mmHg [elevated], and ≥ 90 mmHg [hypertension]), BMI (cutoff from normal Chinese BMI standards: < 18.5 kg/m^2^ [underweight], 18.5–23.9 kg/m^2^ [normal], 24.0–27.9 kg/m^2^ [overweight], and ≥ 28.0 kg/m^2^ [obesity]), drinking status, and smoking status.

## 3. Results

### 3.1. Baseline Characteristics of Participants

The demographic characteristics of the 3546 participants included in this study in the Wave 1 investigation are presented in Table 1. The average age of the participants was 57.14 ± 8.69 years, and 48.96% were males. A total of 113 participants eventually developed CSP after a follow‐up of 9 years (2011–2020). Compared to those without CSP, participants with CSP had lower levels of CREA and UA, a higher level of TG, and a higher prevalence of current smoking and agricultural residence. However, mean values of IR‐related indexes, including TyG, FGB, BUN, TC, HDL‐C, LDL‐C, and HBA1C, showed no significant difference between the two groups. In addition, we also provided a baseline description of the missing variables before interpolation, which is similar to the above results (Table S2).

**TABLE 1:** Baseline characteristics of participants.

Variables	Total (*n* = 3546)	Participants without CSP (*n* = 3433)	Participants with CSP (*n* = 113)	Statistic	*p*
TyG index in w1, mean ± SD	8.69 ± 0.67	8.69 ± 0.67	8.87 ± 0.71	*t* = −2.79	**0.005**
FGB (mg/dL)	109.93 ± 35.29	109.82 ± 35.29	113.33 ± 35.38	*t* = −1.04	0.299
TG (mg/dL)	134.24 ± 108.17	133.34 ± 105.03	161.70 ± 177.44	*t* = −2.75	**0.006**
Gender, n (%)				*χ* ^2^ = 23.45	**<** **0.001**
Male	1736 (48.96)	1706 (49.69)	30 (26.55)		
Female	1810 (51.04)	1727 (50.31)	83 (73.45)		
Age (years)	57.14 ± 8.69	57.06 ± 8.67	59.49 ± 8.85	*t* = −2.92	**0.003**
BUN (mg/dL)	15.52 ± 4.27	15.52 ± 4.25	15.57 ± 4.62	*t* = −0.11	0.915
CREA (μmol/L)	0.77 ± 0.18	0.77 ± 0.18	0.72 ± 0.16	*t* = 3.28	**0.001**
TC (mg/dL)	192.86 ± 39.37	192.66 ± 39.12	198.96 ± 46.15	*t* = −1.67	0.095
HDL‐C (mg/dL)	50.30 ± 15.09	50.35 ± 15.09	48.64 ± 15.13	*t* = 1.19	0.235
UA (mg/dL)	4.42 ± 1.23	4.43 ± 1.23	4.08 ± 1.18	*t* = 2.99	**0.003**
SBP (mmHg)	129.25 ± 20.32	129.22 ± 20.27	130.23 ± 21.89	*t* = −0.52	0.602
DBP (mmHg)	75.67 ± 11.91	75.70 ± 11.89	74.76 ± 12.49	*t* = 0.82	0.411
LDL‐C (mg/dL)	116.40 ± 35.04	116.39 ± 35.08	116.81 ± 33.84	*t* = −0.12	0.901
HBA1C (%) (mmol/mol)	5.25 ± 0.79	5.25 ± 0.79	5.39 ± 0.97	*t* = −1.82	0.068
BMI (kg/m^2^)	23.76 ± 3.46	23.75 ± 3.44	24.06 ± 3.94	*t* = −0.94	0.346
Residence, n (%)				*χ* ^2^ = 11.05	**<** **0.001**
Agricultural residence	2907 (81.98)	2801 (81.59)	106 (93.81)		
Non‐agricultural residence	639 (18.02)	632 (18.41)	7 (6.19)		
Marriage status, n (%)				*χ* ^2^ = 0.44	0.508
Out of marriage	286 (8.07)	275 (8.01)	11 (9.73)		
In marriage	3260 (91.93)	3158 (91.99)	102 (90.27)		
Education level, n (%)				*χ* ^2^ = 23.06	**<** **0.001**
Primary school or lower	2254 (63.56)	2158 (62.86)	96 (84.96)		
Secondary school or higher	1292 (36.44)	1275 (37.14)	17 (15.04)		
Drinking status, n (%)				*χ* ^2^ = 2.02	0.155
No	2157 (60.83)	2081 (60.62)	76 (67.26)		
Yes	1389 (39.17)	1352 (39.38)	37 (32.74)		
Smoking status, n (%)				*χ* ^2^ = 4.47	**0.034**
No	2474 (69.77)	2385 (69.47)	89 (78.76)		
Yes	1072 (30.23)	1048 (30.53)	24 (21.24)		

*Note:* Continuous variables were expressed as mean ± standard deviation (SD) in the case of normal distribution and compared between two groups by the ANOVA test. If the count variable had a theoretical number < 10, Fisher’s exact probability test was used. Categorical variables are presented as counts (percentages) and compared by the chi‐square test. FGB: fasting blood glucose; TG: triglyceride; TC: serum total cholesterol; HbA1c: hemoglobin A1C; TyG: triglyceride–glucose; Cum TyG: cumulative TyG. The bold values mean that the *p* values have a statistical difference below 0.05.

Abbreviations: BMI, body mass index; BUN, blood urea nitrogen; CSP, chronic severe pain; DBP, diastolic blood pressure; HDL‐C, high‐density lipoprotein cholesterol; LDL‐C, low‐density lipoprotein cholesterol; SBP, systolic blood pressure; UA, uric acid.

### 3.2. Association Between Baseline TyG Index and CSP Incidence

To investigate the association between baseline TyG index and CSP, we conducted three covariate models with different adjustments for logistic regression analyses (Table 2). In univariate binary logistic regression (crude model), the ORs (95% CIs) for incident CSP were 1.43 (1.11, 1.83) with *p* = 0.005. In multivariable logistic regression (Model II), which adjusted for various confounding factors, the ORs (95% CIs) for incident CSP were 1.51 (1.11, 1.96) with *p* = 0.020. We assessed multicollinearity using the variance inflation factor (VIF). All generalized VIF (GVIF) values (GVIF^(1/(2∗Df))^) were below 1.5, indicating no substantial multicollinearity among the covariates in the model. These results indicate that the baseline TyG index has a significant correlation with high CSP incidence.

**TABLE 2:** Odds ratios for incident chronic severe pain occurring in different logistic regression models with the TyG index.

Variables	Crude	Model I	Model II
*β*	0.35	0.34	0.41
S.E	0.13	0.13	0.13
*Z*	2.79	2.54	3.06
*p*	0.005	0.011	0.020
OR (95% CI)	1.43 (1.11∼1.83)	1.40 (1.08∼1.82)	1.51 (1.16∼1.96)

*Note:* Model I, adjusted for age and gender. Model II, adjusted for age, gender, education, marital status, residence, smoking status, drinking status, uric acid, and blood urea nitrogen.

**FIGURE 3:**
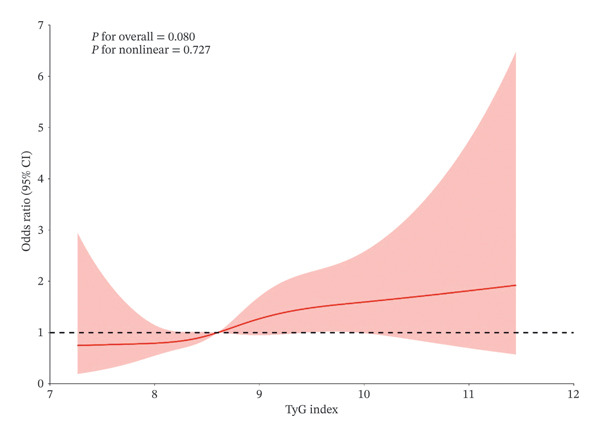
Cubic model of the association between baseline TyG index and CSP occurs after adjustment for covariates.

The restricted cubic spline regression model is shown in Figure 3. The association between the TyG index and CSP risk is linear with a cutoff of TyG = 8.602, and the indicator of a higher TyG index corresponds to higher ORs of CSP.

In a supporting analysis shown in Table S3 that categorized participants using a data‐driven threshold from the restricted cubic spline cutoff (TyG = 8.602), the high TyG group remained associated with a significantly increased risk of CSP (OR = 1.80, 95% CI: 1.21–2.69), corroborating the findings from the continuous variable analysis.

Sensitivity analyses using alternative, single‐wave definitions of severe pain did not yield significant associations (data not shown), supporting the specificity of our primary finding to persistent pain.

### 3.3. TyG Index‐Based Group Classification

**FIGURE 4:**
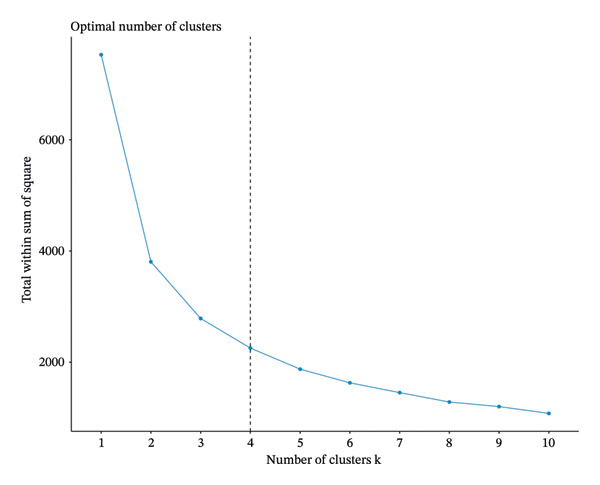
K‐means clustering method for clustering the triglyceride–glucose index.

**FIGURE 5:**
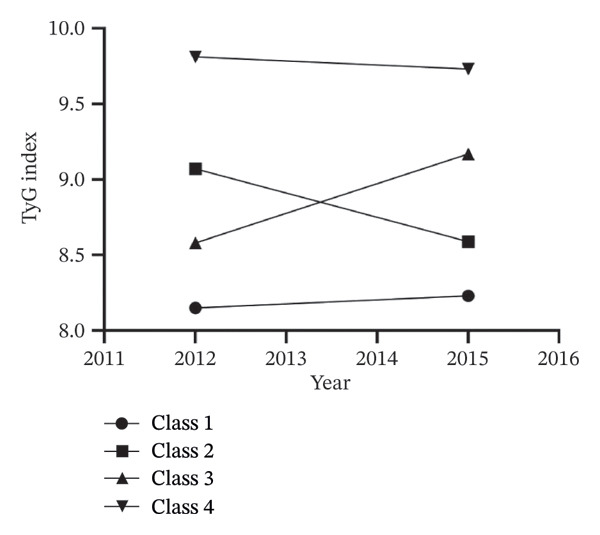
The triglyceride–glucose index trend by k‐means clustering.

IR is a chronic disease for which long‐term evaluation and therapy are necessary for controlling the incidence of IR‐related systemic diseases. Thus, compared to a one‐time TyG index observation, multiple dynamic evaluations of the index might better represent the progression and severity of IR. We further included participants’ TyG index in both Wave 1 and Wave 3 for further investigation. The k‐means clustering method is used for TyG index‐based group classification due to its simplicity and scalability [17]. In this study, when clustering participants into 4 classes, k‐means clustering works best (shown in Figure 4) [16–18]. For Class 1, TyG ranged from 8.14 to 8.23, representing the best TyG level control. For Class 2, TyG ranged from 9.07 to 8.63, representing a decrease in TyG levels from a high level to a low level with better control. For Class 3, TyG ranged from 8.60 to 9.17, representing an increase in TyG levels from a low level to a high level with poor control. For Class 4, TyG ranged from 9.82 to 9.74, representing the worst control of TyG level (shown in Figure 5).

The baseline characteristics of the participants after the k‐means clustering method were shown in Table 3. Compared to Class 1, participants in other classes had higher levels of cumulative TyG index, FGB, TG, TC, UA, SBP, DBP, HBA1C, and BMI, but lower levels of BUN and HDL‐C, and lower prevalence of smoking and drinking status. In addition, we also provided a baseline description of the missing variables before interpolation, which is similar to the above results (Table S4).

**TABLE 3:** Baseline characteristics of participants in different classes.

Variables	Total (*n* = 3546)	Class 1 (*n* = 1395)	Class 2 (*n* = 826)	Class 3 (*n* = 851)	Class 4 (*n* = 474)	Statistic	*p*
TyG index in w1, mean ± SD	8.69 ± 0.67	8.15 ± 0.31	9.07 ± 0.33	8.59 ± 0.34	9.81 ± 0.59	*F* = 2736.31	**<** **0.001**
TyG index in w3, mean ± SD	8.74 ± 0.63	8.23 ± 0.32	8.59 ± 0.30	9.17 ± 0.32	9.73 ± 0.50	*F* = 2809.30	**<** **0.001**
Cum. TyG, Tyg‐year	34.86 ± 2.31	32.76 ± 0.92	35.33 ± 0.94	35.52 ± 1.00	39.06 ± 1.54	*F* = 4591.63	**<** **0.001**
CSP incidence	113 (3.19%)	34 (2.44%)	32 (3.87%)	22 (2.59%)	25 (5.27%)	*χ* ^2^ = 11.50	**0.009**
FGB (mg/dL)	109.93 ± 35.29	98.00 ± 13.77	113.80 ± 29.76	103.93 ± 18.13	149.10 ± 67.75	*F* = 332.06	**<** **0.001**
TG (mg/dL)	134.24 ± 108.17	74.58 ± 21.74	165.39 ± 61.37	110.40 ± 35.74	298.36 ± 192.94	*F* = 999.78	**<** **0.001**
Gender, n (%)						*χ* ^2^ = 26.42	**<** **0.001**
Male	1816 (48.22)	788 (53.42)	393 (44.66)	407 (44.68)	228 (45.60)		
Female	1950 (51.78)	687 (46.58)	487 (55.34)	504 (55.32)	272 (54.40)		
Age (years)	57.14 ± 8.69	57.15 ± 8.62	58.01 ± 8.89	56.68 ± 8.56	56.42 ± 8.67	*F* = 4.64	**0.003**
BUN (mg/dL)	15.52 ± 4.27	15.79 ± 4.44	14.98 ± 4.12	15.58 ± 4.19	15.57 ± 4.03	*F* = 6.33	**<** **0.001**
CREA (μmol/L)	0.77 ± 0.18	0.76 ± 0.17	0.77 ± 0.18	0.77 ± 0.17	0.79 ± 0.21	*F* = 3.49	**0.015**
TC (mg/dL)	192.86 ± 39.37	181.49 ± 34.26	197.91 ± 39.36	196.71 ± 36.49	210.63 ± 47.90	*F* = 83.67	**<** **0.001**
HDL‐C (mg/dL)	50.30 ± 15.09	56.73 ± 14.77	46.78 ± 13.13	50.54 ± 13.71	37.10 ± 10.30	*F* = 270.62	**<** **0.001**
UA (mg/dL)	4.42 ± 1.23	4.23 ± 1.16	4.49 ± 1.26	4.46 ± 1.15	4.79 ± 1.37	*F* = 27.54	**<** **0.001**
SBP (mmHg)	129.25 ± 20.32	126.07 ± 19.89	131.01 ± 20.80	129.56 ± 19.96	134.97 ± 19.76	*F* = 26.63	**<** **0.001**
DBP (mmHg)	75.67 ± 11.91	73.68 ± 11.62	76.33 ± 12.22	76.15 ± 11.54	79.48 ± 11.77	*F* = 31.21	**<** **0.001**
LDL‐C (mg/dL)	116.40 ± 35.04	112.04 ± 30.13	118.66 ± 36.56	126.01 ± 34.26	108.04 ± 42.24	*F* = 39.95	**<** **0.001**
HBA1C (%) (mmol/mol)	5.25 ± 0.79	5.08 ± 0.44	5.26 ± 0.70	5.19 ± 0.60	5.88 ± 1.46	*F* = 137.82	**<** **0.001**
BMI (kg/m^2^)	23.76 ± 3.46	22.72 ± 3.16	24.15 ± 3.36	24.16 ± 3.47	25.41 ± 3.50	*F* = 91.99	**<** **0.001**
Residence, n (%)						*χ* ^2^ = 14.57	**0.002**
Agricultural residence	2907 (81.98)	1173 (84.09)	688 (83.29)	679 (79.79)	367 (77.43)		
Non‐agricultural residence	639 (18.02)	222 (15.91)	138 (16.71)	172 (20.21)	107 (22.57)		
Marriage status, n (%)						*χ* ^2^ = 10.07	**0.018**
Out of marriage	286 (8.07)	122 (8.75)	81 (9.81)	54 (6.35)	29 (6.12)		
In marriage	3260 (91.93)	1273 (91.25)	745 (90.19)	797 (93.65)	445 (93.88)		
Education level, n (%)						*χ* ^2^ = 3.09	0.378
Primary school or lower	2254 (63.56)	907 (65.02)	528 (63.92)	525 (61.69)	294 (62.03)		
Secondary school or higher	1292 (36.44)	488 (34.98)	298 (36.08)	326 (38.31)	180 (37.97)		
Drinking status, n (%)						*χ* ^2^ = 3.86	0.277
No	2157 (60.83)	830 (59.50)	522 (63.20)	525 (61.69)	280 (59.07)		
Yes	1389 (39.17)	565 (40.50)	304 (36.80)	326 (38.31)	194 (40.93)		
Smoking status, n (%)						*χ* ^2^ = 12.86	**0.005**
No	2474 (69.77)	929 (66.59)	603 (73.00)	597 (70.15)	345 (72.78)		
Yes	1072 (30.23)	466 (33.41)	223 (27.00)	254 (29.85)	129 (27.22)		

*Note:* Continuous variables were expressed as mean ± standard deviation (SD) in the case of normal distribution and compared between two groups by the ANOVA test. If the count variable had a theoretical number < 10, Fisher’s exact probability test was used. Categorical variables are presented as counts (percentages) and compared by chi‐square test. FGB: fasting blood glucose; TG: triglyceride; TC: serum total cholesterol; HbA1c: hemoglobin A1C; TyG: triglyceride–glucose; Cum TyG: cumulative TyG. The bold values mean that the *p* values have a statistical difference below 0.05.

Abbreviations: BMI, body mass index; BUN, blood urea nitrogen; CSP, chronic severe pain; DBP, diastolic blood pressure; HDL‐C, high‐density lipoprotein cholesterol; LDL‐C, low‐density lipoprotein cholesterol; SBP, systolic blood pressure; UA, uric acid.

### 3.4. Association Between TyG Control Level and CSP Incidence

Next, we conducted the logistic regression analyses between different TyG control levels and CSP (Table 4). Three covariate models were developed with varying adjustments to capture and represent this correlation: Crude model for the univariate binary logistic regression; Model I for adjustment from age, gender and and the baseline TyG index; and Model II for adjustment from all ascertainment of covariates. In the crude model, compared to Class 1, the ORs for CSP were 1.61 (0.99, 2.63) with *p* = 0.056 for Class 2, 1.06 (0.62, 1.83) with *p* = 0.827 for Class 3, and 2.23 (1.32, 3.78) with *p* = 0.003 for Class 4. After adjusting for age and gender in Model I or adjusting for all covariates in Model II, the ORs for CSP in Class 4 were still significantly higher than Class 1 (2.30 (1.01, 5.25) with *p* = 0.048 in Model II). We assessed multicollinearity using the VIF. All GVIF values (GVIF^(1/(2∗Df))^) were below 3, indicating no substantial multicollinearity among the covariates in the model.

**TABLE 4:** Odds ratios for incident chronic severe pain occurring in different logistic regression models.

Cluster	Case	Crude	Model I	Model II
Total	113 (3.19%)			
Class 1	34 (2.44%)	1.00 (Reference)	1.00 (Reference)	1.00 (Reference)
Class 2	32 (3.87%)	1.61 (0.99∼2.63) 0.056	1.29 (0.73∼2.28) 0.376	1.39 (0.78∼2.46) 0.263
Class 3	22 (2.59%)	1.06 (0.62∼1.83) 0.827	1.23 (0.78∼1.95) 0.374	1.35 (0.85∼2.15) 0.201
Class 4	25 (5.27%)	2.23 (1.32∼3.78) 0.003	2.12 (0.94∼4.81) 0.072	2.30 (1.01∼5.25) 0.048

*Note:* Model I, adjusted for age, gender, and the baseline TyG index. Model II, adjusted for age, gender, education, marital status, residence, smoking status, drinking status, uric acid, blood urea nitrogen, and the baseline TyG index.

**FIGURE 6:**
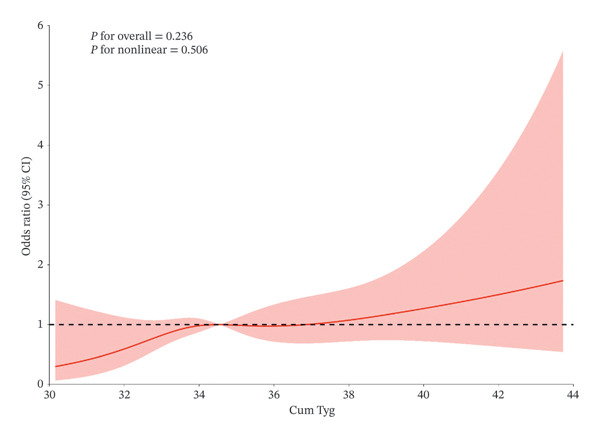
Cubic model of the association between cumulative TyG index and CSP occurs after adjustment for covariates.

Moreover, the restricted cubic spline regression model is shown in Figure 6. The association between the cumulative TyG index and CSP risk is linear. A higher cumulative TyG index corresponds to higher ORs of CSP. These results indicated that worse control of the TyG index has a significant correlation with higher CSP incidence.

### 3.5. Subgroup Analyses

To further explore the relationship between TyG control level and the risk of incident CSP events, we conducted subgroup analyses stratified by potential risk factors. As shown in Table S5 and Figure S1, people with the worst control of TyG in years (from Class 4) showed a higher risk of CSP in the age of 45–65, female, lower education level, married, agriculture residence location, no smoking nor drinking, and moderate blood pressure (SBP 90–140 mmHg, DBP 60–90 mmHg) and BMI (18.5–23.9 kg/m^2^) subgroups compared to their counterparts. Critically, none of the interaction terms were statistically significant (all *p* for interaction > 0.05).

## 4. Discussion

Herein, we revealed a significant linear association between the TyG control level and future CSP incidence in Chinese participants. To our knowledge, this is the first large‐scale study to demonstrate that the TyG and the worst control of TyG (Class 4) were strong predictors of CSP incidence.

Accumulating studies emphasized the predictive value of the TyG index for IR [8]. Compared to the classical IR diagnostic approach, HOMA‐IR, the TyG index is a much simpler method without requiring the use of insulin assays [19, 20]. The TyG index is calculated by blood triglycerides and glucose. Thus, abnormal alteration of the TyG index reflects both lipid and glucose metabolism disorders. Recent studies offered evidence to regard the TyG index as an ideal biochemical indicator of lipid and glucose metabolic‐related diseases, such as atherosclerosis and coronary artery disease [21, 22], stroke [23], and type 2 diabetes [24, 25]. It is worth noticing that lipid and glucose metabolism disorders are also crucial pathogenesis of chronic pain [26–28]. On the one hand, a number of membrane lipid‐derived mediators play pivotal roles in the initiation, maintenance, and regulation of various types of acute and chronic pain [29]. On the other hand, chronic pain at multiple painful sites is more prevalent among diabetics than among nondiabetics [30]. Therefore, regarding the TyG index as a predictor of CSP incidence has sufficient theoretical basis and clinical implication.

As a chronic outcome, the development of CSP is more plausibly linked to sustained metabolic dysregulation. The long‐term TyG trajectory may provide a more appropriate measure of the relevant exposure than a single assessment [31, 32]. This study conducted logistic regression analyses of both the baseline TyG index in the Wave 1 investigation and the cumulative TyG index in the Wave 1 and Wave 3 investigations with CSP incidence. In results, the baseline TyG index was associated with CSP incidence during the 9‐year follow‐up period. This association was robust, showing a linear dose–response relationship and remaining significant when using a data‐driven threshold (TyG = 8.602) for risk stratification. The consistency across methods underscores that a single baseline TyG measurement is a potent predictor, highlighting the value of early metabolic health assessment for preventing chronic pain.

Further, after including the TyG index in Wave 3 and classifying participants according to their TyG control level, it was found that the class with the worst TyG control had a significantly increased ORs of CSP incidence, and the 3‐year cumulative TyG index was linearly associated with the ORs of CSP incidence. These findings suggest that both one‐time analyzing of the TyG index and its trajectory are suitable for predicting CSP incident risk, and long‐term well control of the TyG index may be an efficient approach to reduce the risk of CSP incidence.

Besides, the finding of similar CSP risks for improving (Class 2) and worsening (Class 3) trajectories, as opposed to the high risk in the persistently high trajectory (Class 4), can be explained through two complementary theoretical lenses. First, from a cumulative burden perspective, the net metabolic insult over time may be comparable between Class 2 (high‐to‐low) and Class 3 (low‐to‐high), as the early high exposure in the former and the later high exposure in the latter could result in a similar overall dose, which was insufficient to significantly elevate risk compared to the sustained, high‐grade exposure in Class 4. Second, a threshold effect may also be at play, wherein the transient crossings of a risk threshold in the fluctuating trajectories (Classes 2 and 3) are less pathogenic than the stable, long‐term maintenance above that critical threshold seen in Class 4. Consequently, the primary driver of CSP risk appears to be the severity and chronicity of exposure that defines a stable dysmetabolic state, rather than short‐term directional changes. This insight underscores that the clinical focus should be on identifying and managing persistent dysmetabolism.

Collinearity diagnostics confirmed the robustness of our models, with all GVIF values below 3, indicating no substantial multicollinearity among the covariates.

The distribution of the TyG index varies according to the demographic characteristics of the subjects, such as age and sex, making it difficult to estimate the optimal cutoff point [33, 34]. Thus, the relationship between TyG control level and the risk of incident CSP events should be further investigated in different populations. In the subgroup analyses, the interactions were not significant in all kinds of subgroups. The lack of significant effect modification supports the generalizability of the association between unfavorable TyG trajectories and CSP risk, suggesting it may be applicable across a wide spectrum of middle‐aged and older adults [35, 36]. Notably, people with the worst control of TyG in years (Class 4) showed a higher risk of CSP in populations of age 45–65, female, lower education level, married, agriculture residence, no smoking nor drinking, moderate blood pressure, and moderate BMI level, which suggests that the findings of the present study may be more applicable to the vast majority of the population.

This study has several important limitations inherent to its observational design and data availability. First, despite adjustments for a comprehensive set of confounders, residual confounding from unmeasured or imprecisely measured factors cannot be entirely ruled out. Notably, the CHARLS survey lacks detailed data on specific pain medications, targeted treatments for CSP, and objective measures of physical activity—all of which could act as either confounders or mediators. Furthermore, we were unable to account for other potential organic causes of CSP, such as specific traumatic injuries or surgical histories, which might influence the observed relationship. Second, our operational definition of CSP, while a methodological strength for identifying a severe and persistent pain phenotype with high specificity, inherently focuses the study on a distinct clinical subgroup. Consequently, while the internal validity for this phenotype is enhanced, the generalizability of our findings may be strongest for this severe subtype and might not directly extend to individuals with milder or more fluctuating forms of chronic pain. Furthermore, this definition is constrained by the structure of the CHARLS database, which did not collect more detailed, clinically validated pain intensity measures. This limitation in pain characterization is an inherent challenge of the secondary data analysis.

Third, the statistical power of our analyses, particularly the subgroup analyses, was constrained by the relatively low incidence of CSP in the refined cohort. This limitation warrants caution in the interpretation of nonsignificant findings, as the study may be underpowered to detect modest but clinically meaningful associations or interaction effects within specific population strata. Finally, the requirement for complete TyG index data at both baseline and follow‐up waves led to the exclusion of some participants, introducing a potential for selection bias. If the missingness of biomarker data were associated with poorer health status (and thus potentially higher TyG and higher pain risk), our results could represent a conservative estimate of the true association. These limitations underscore the value of future research utilizing larger, more comprehensively phenotyped cohorts or pooled datasets to validate our findings and explore these associations with greater precision across diverse subpopulations.

## 5. Conclusion

In this study, we discovered that the TyG index should be regarded as a simple indicator of CSP incidence in the general population. Continuous monitoring of the TyG index is also suitable for predicting CSP incident risk, and long‐term well control of the TyG index may be an efficient approach to reduce the risk of CSP incidence.

## Funding

This study was supported by the Shenzhen SMART fund, C2301006.

## Conflicts of Interest

The authors declare no conflicts of interest.

## Supporting Information

Additional supporting information can be found online in the Supporting Information section.

## Supporting information


**Supporting Information 1** Table S1 Missing number for variables.

**Supporting Information 2** Table S2 Baseline Characteristics of missing variables before multiple imputation.

**Supporting Information 3** Table S3 Odds ratios for incident chronic severe pain occurring in different logistic regression models with high‐TyG class.

**Supporting Information 4** Table S4. Baseline Characteristics of missing variables before multiple imputations in different classes.

**Supporting Information 5** Table S5. Subgroup analysis of the associations between different classes and chronic severe pain incidence.

**Supporting Information 6** Figure S1. Subgroup analysis of the associations between different classes and chronic severe pain incidence.

## Data Availability

The data that support the findings of this study are available on request from the corresponding author. The data are not publicly available due to privacy or ethical restrictions.
