# Supplementary material for: Association Between the Triglyceride–Glucose Index and Incident Chronic Severe Pain in Middle‐Aged and Older Chinese Adults: A Nationwide Cohort Study
Source: Pain Res Manag. 2026 Jan 30;2026:2464060. doi: 10.1155/prm/2464060 (PMC12856695; doi:10.1155/prm/2464060)
Supplement: Supplementary file 1 — Supporting Information 1 Table S1 Missing number for variables. [file PRM-2026-2464060-s006.docx]

**Table S1 Missing number for variables.**

| **Variables** | **Missing number (%)** |
| --- | --- |
| Age | 0 (0) |
| Gender | 0 (0) |
| Education level | 0 (0) |
| Current married | 0 (0) |
| Residence | 0 (0) |
| Smoking | 39 (1.10%) |
| Drinking | 2 (0.05%) |
| SBP | 409 (11.53%) |
| DBP | 411 (11.59%) |
| FBG | 0 (0) |
| TG | 0 (0) |
| BUN | 0 (0) |
| TC | 0 (0) |
| HDL-C | 0 (0) |
| LDL-C | 6 (0.17%) |
| CRP | 0 (0) |
| HbA1c | 21 (0.54%) |
| UA | 0 (0) |
| BMI | 403(11.36%) |

Abbreviations: SBP: systolic blood pressure; DBP: diastolic blood pressure; BMI: body mass index; FBG: fasting blood-glucose; TG: Triglycerides; BUN: blood urea nitrogen; TC: Total Cholesterol; HDL-C: high-density lipoprotein cholesterol; LDL-C: low-density lipoprotein cholesterol; CRP: C-reactive protein; HbA1c: [hemoglobin A1C](http://www.dictall.com/indu/214/213618925C9.htm); UA: uric acid.
