# Supplementary material for: Association Between the Triglyceride–Glucose Index and Incident Chronic Severe Pain in Middle‐Aged and Older Chinese Adults: A Nationwide Cohort Study
Source: Pain Res Manag. 2026 Jan 30;2026:2464060. doi: 10.1155/prm/2464060 (PMC12856695; doi:10.1155/prm/2464060)
Supplement: Supplementary file 2 — Supporting Information 2 Table S2 Baseline Characteristics of missing variables before multiple imputation. [file PRM-2026-2464060-s004.docx]

**Table S2Baseline Characteristics of missing variables before multiple interpolation.**

| Variables | Total | Participants without CSP | Participants with CSP | Statistic | *P* |
| --- | --- | --- | --- | --- | --- |
|  |  |  |  |  |  |
| SBP,mmHg | 129.25 ± 20.32 | 129.22 ± 20.27 | 130.23 ± 21.89 | t=-0.52 | 0.602 |
| DBP,mmHg | 75.67 ± 11.91 | 75.70 ± 11.89 | 74.76 ± 12.49 | t=0.82 | 0.411 |
| LDL-C, mg/dL | 116.40 ± 35.04 | 116.39 ± 35.08 | 116.81 ± 33.84 | t=-0.12 | 0.901 |
| HBA1C, % (mmol/mol) | 5.25 ± 0.79 | 5.25 ± 0.79 | 5.39 ± 0.97 | t=-1.82 | 0.068 |
| BMI, kg/m2 | 23.76 ± 3.46 | 23.75 ± 3.44 | 24.06 ± 3.94 | t=-0.94 | 0.346 |
| Drinking status, n(%) |  |  |  | χ²=2.02 | 0.155 |
| No | 2157 (60.83) | 2081 (60.62) | 76 (67.26) |  |  |
| Yes | 1389 (39.17) | 1352 (39.38) | 37 (32.74) |  |  |
| Smoking status, n(%) |  |  |  | χ²=4.47 | **0.034** |
| No | 2474 (69.77) | 2385 (69.47) | 89 (78.76) |  |  |
| Yes | 1072 (30.23) | 1048 (30.53) | 24 (21.24) |  |  |

Abbreviations:SBP:systolic blood pressure; DBP: diastolic blood pressure; BMI: body mass index; LDL-C: low-density lipoprotein cholesterol; HbA1c: [hemoglobin A1C](http://www.dictall.com/indu/214/213618925C9.htm).

Notes: Continuous variables were expressed as mean±standard deviation (SD) in case of normal distribution and compared between two groups by ANOVA test. If the count variable had a theoretical number<10, Fisher’s exact probability test was used. Categorical variables are presented as counts (percentages) and compared by Chi-square test
