# Supplementary material for: Association Between the Triglyceride–Glucose Index and Incident Chronic Severe Pain in Middle‐Aged and Older Chinese Adults: A Nationwide Cohort Study
Source: Pain Res Manag. 2026 Jan 30;2026:2464060. doi: 10.1155/prm/2464060 (PMC12856695; doi:10.1155/prm/2464060)
Supplement: Supplementary file 3 — Supporting Information 3 Table S3 Odds ratios for incident chronic severe pain occurring in different logistic regression models with high‐TyG class. [file PRM-2026-2464060-s002.docx]

| Variables | Crude | Model Ⅰ | Model Ⅱ |
| --- | --- | --- | --- |
|  |  |  |  |
|  |  |  |  |
|  |  |  |  |
| β | 0.54 | 0.46 | 0.59 |
| S.E | 0.20 | 0.20 | 0.20 |
| Z | 2.74 | 2.30 | 2.88 |
| *P* | 0.006 | 0.022 | 0.004 |
| OR (95%CI) | 1.72 (1.17 ~ 2.53) | 1.58 (1.07 ~ 2.33) | 1.80 (1.21 ~ 2.69) |

**Table S3 Odds ratios for incident chronic severe pain occurs in different logistic regression model with high-TyG class**

Model I, adjusted for age, gender.

Model II, adjusted for age, gender, education, marital status, residence, smoking status, drinking status, uric acid, blood urea nitrogen.
