# Supplementary material for: Association Between the Triglyceride–Glucose Index and Incident Chronic Severe Pain in Middle‐Aged and Older Chinese Adults: A Nationwide Cohort Study
Source: Pain Res Manag. 2026 Jan 30;2026:2464060. doi: 10.1155/prm/2464060 (PMC12856695; doi:10.1155/prm/2464060)
Supplement: Supplementary file 4 — Supporting Information 4 Table S4. Baseline Characteristics of missing variables before multiple imputations in different classes. [file PRM-2026-2464060-s003.docx]

**Table S4Baseline Characteristics of missing variables before multiple interpolation in different classes.**

| Variables | Total | Class1 | Class2 | Class3 | Class4 | Statistic | *P* |
| --- | --- | --- | --- | --- | --- | --- | --- |
|  |  |  |  |  |  |  |  |
| SBP,mmHg | 129.25 ± 20.32 | 126.07 ± 19.89 | 131.01 ± 20.80 | 129.56 ± 19.96 | 134.97 ± 19.76 | F=26.63 | **<.001** |
| DBP,mmHg | 75.67 ± 11.91 | 73.68 ± 11.62 | 76.33 ± 12.22 | 76.15 ± 11.54 | 79.48 ± 11.77 | F=31.21 | **<.001** |
| LDL-C, mg/dL | 116.40 ± 35.04 | 112.04 ± 30.13 | 118.66 ± 36.56 | 126.01 ± 34.26 | 108.04 ± 42.24 | F=39.95 | **<.001** |
| HBA1C, % (mmol/mol) | 5.25 ± 0.79 | 5.08 ± 0.44 | 5.26 ± 0.70 | 5.19 ± 0.60 | 5.88 ± 1.46 | F=137.82 | **<.001** |
| BMI, kg/m2 | 23.76 ± 3.46 | 22.72 ± 3.16 | 24.15 ± 3.36 | 24.16 ± 3.47 | 25.41 ± 3.50 | F=91.99 | **<.001** |
| Drinking status, n(%) |  |  |  |  |  | χ²=3.86 | 0.277 |
| No | 2157 (60.83) | 830 (59.50) | 522 (63.20) | 525 (61.69) | 280 (59.07) |  |  |
| Yes | 1389 (39.17) | 565 (40.50) | 304 (36.80) | 326 (38.31) | 194 (40.93) |  |  |
| Smoking status, n(%) |  |  |  |  |  | χ²=12.86 | **0.005** |
| No | 2474 (69.77) | 929 (66.59) | 603 (73.00) | 597 (70.15) | 345 (72.78) |  |  |
| Yes | 1072 (30.23) | 466 (33.41) | 223 (27.00) | 254 (29.85) | 129 (27.22) |  |  |

Abbreviations: SBP:systolic blood pressure; DBP: diastolic blood pressure; BMI: body mass index; LDL-C: low-density lipoprotein cholesterol; HbA1c: [hemoglobin A1C](http://www.dictall.com/indu/214/213618925C9.htm).

Notes: Continuous variables were expressed as mean±standard deviation (SD) in case of normal distribution and compared between two groups by ANOVA test. If the count variable had a theoretical number<10, Fisher’s exact probability test was used. Categorical variables are presented as counts (percentages) and compared by Chi-square test
