# Supplementary material for: Association Between the Triglyceride–Glucose Index and Incident Chronic Severe Pain in Middle‐Aged and Older Chinese Adults: A Nationwide Cohort Study
Source: Pain Res Manag. 2026 Jan 30;2026:2464060. doi: 10.1155/prm/2464060 (PMC12856695; doi:10.1155/prm/2464060)
Supplement: Supplementary file 5 — Supporting Information 5 Table S5. Subgroup analysis of the associations between different classes and chronic severe pain incidence. [file PRM-2026-2464060-s005.docx]

**Table S5.Subgroup analysis of the associations between different classes and chronic severe pain incidence.**

|  | **Case** | **TyG out of control** | | **OR (95%CI)** | ***P*** | ***P* for interaction** |
| --- | --- | --- | --- | --- | --- | --- |
|  |  | **No** | **Yes** |  |  |  |
| All patients | 3546 | 88/3072 | 25/474 | 1.89 (1.20 ~ 2.98) | **0.006** |  |
| Age, years |  |  |  |  |  | 0.964 |
| 45-65 | 2851 | 63/2458 | 19/393 | 1.93 (1.14 ~ 3.26) | **0.014** |  |
| ≥65 | 695 | 25/614 | 6/81 | 1.88 (0.75 ~ 4.74) | 0.178 |  |
| Gender |  |  |  |  |  | 0.746 |
| Male | 1736 | 23/1513 | 7/223 | 2.10 (0.89 ~ 4.95) | 0.090 |  |
| Female | 1810 | 65/1559 | 18/251 | 1.78 (1.03 ~ 3.05) | **0.037** |  |
| Education level |  |  |  |  |  | 0.524 |
| Primary school or lower | 2254 | 74/1960 | 22/294 | 2.06 (1.26 ~ 3.37) | **0.004** |  |
| Secondary school or higher | 1292 | 14/1112 | 3/180 | 1.33 (0.38 ~ 4.67) | 0.657 |  |
| Marriage status |  |  |  |  |  | 0.975 |
| No | 286 | 11/257 | 0/29 | 0.00 (0.00 ~ Inf) | 0.990 |  |
| Yes | 3260 | 77/2815 | 25/445 | 2.12 (1.33 ~ 3.36) | **0.001** |  |
| Residence |  |  |  |  |  | 0.824 |
| Agriculture | 2907 | 40/1481 | 66/1426 | 1.75 (1.17 ~ 2.61) | **0.006** |  |
| Others | 639 | 2/292 | 5/347 | 2.12 (0.41 ~ 11.01) | 0.371 |  |
| Smoking status |  |  |  |  |  | 0.260 |
| No | 2155 | 25/1057 | 51/1098 | 2.01 (1.24 ~ 3.27) | **0.005** |  |
| Yes | 1388 | 17/715 | 20/673 | 1.26 (0.65 ~ 2.42) | 0.493 |  |
| Drinkingstatus |  |  |  |  |  | 0.641 |
| No | 2447 | 68/2129 | 21/345 | 1.96 (1.19 ~ 3.25) | **0.009** |  |
| Yes | 1059 | 20/943 | 4/129 | 1.48 (0.50 ~ 4.39) | 0.483 |  |
| SBP, mmHg |  |  |  |  |  | 0.502 |
| ＜90 | 23 | 0/22 | 0/1 | 1.00 (0.00 ~ Inf) | 1.000 |  |
| 90-140 | 2599 | 62/2292 | 18/307 | 2.24 (1.31 ~ 3.84) | **0.003** |  |
| ＞140 | 924 | 26/758 | 7/166 | 1.24 (0.53 ~ 2.91) | 0.621 |  |
| DBP, mmHg |  |  |  |  |  | 0.799 |
| ＜60 | 267 | 8/247 | 1/20 | 1.57 (0.19 ~ 13.24) | 0.677 |  |
| 60-90 | 2853 | 69/2490 | 20/363 | 2.05 (1.23 ~ 3.41) | **0.006** |  |
| ＞90 | 426 | 11/335 | 4/91 | 1.35 (0.42 ~ 4.36) | 0.611 |  |
| BMI,kg/m^2^ |  |  |  |  |  | 0.827 |
| ＜18.5 | 139 | 4/133 | 0/6 | 0.00 (0.00 ~ Inf) | 0.995 |  |
| 18.5-23.9 | 1892 | 49/1720 | 10/172 | 2.11 (1.05 ~ 4.23) | **0.037** |  |
| 24-27.9 | 1110 | 24/917 | 8/193 | 1.61 (0.71 ~ 3.64) | 0.253 |  |
| ≥28 | 405 | 11/302 | 7/103 | 1.93 (0.73 ~ 5.12) | 0.187 |  |

Abbreviations:SBP: systolic blood pressure; DBP: diastolic blood pressure; BMI:body mass index.

Notes: In addition to the stratification variables themselves, age, gender, education, marital status, Hukou, smoking status, drinking status, SBP,DBP and BMI were adjusted.
