# Supplementary material for: Association Between the Triglyceride–Glucose Index and Incident Chronic Severe Pain in Middle‐Aged and Older Chinese Adults: A Nationwide Cohort Study
Source: Pain Res Manag. 2026 Jan 30;2026:2464060. doi: 10.1155/prm/2464060 (PMC12856695; doi:10.1155/prm/2464060)
Supplement: Supplementary file 6 — Supporting Information 6 Figure S1. Subgroup analysis of the associations between different classes and chronic severe pain incidence. [file PRM-2026-2464060-s001.docx]

**Figure S1. Subgroup analysis of the associations between different classes and chronic severe pain incidence.**


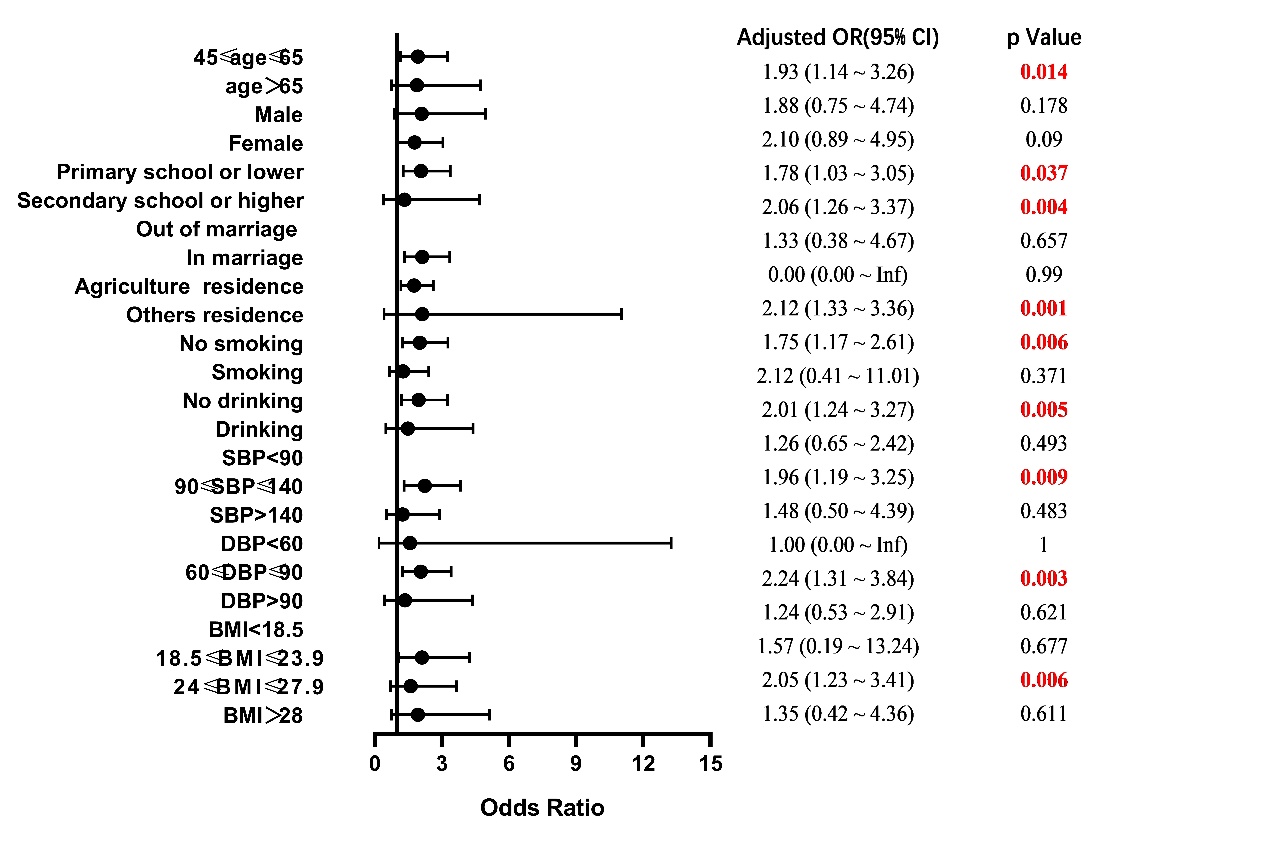


Abbreviations: SBP: systolic blood pressure; DBP: diastolic blood pressure; BMI: body mass index.

Notes: In addition to the stratification variables themselves, age, gender, education, marital status, Residence, smoking status, drinking status, SBP, DBP and BMI were adjusted.
